# Activation of Adhesion GPCR EMR2/ADGRE2 Induces Macrophage Differentiation and Inflammatory Responses *via* Gα_16_/Akt/MAPK/NF-κB Signaling Pathways

**DOI:** 10.3389/fimmu.2017.00373

**Published:** 2017-04-03

**Authors:** Kuan-Yu I, Yi-Shu Huang, Ching-Hsun Hu, Wen-Yi Tseng, Chia-Hsin Cheng, Martin Stacey, Siamon Gordon, Gin-Wen Chang, Hsi-Hsien Lin

**Affiliations:** ^1^Department of Microbiology and Immunology, College of Medicine, Chang Gung University, Taoyuan, Taiwan; ^2^Division of Rheumatology, Allergy and Immunology, Chang Gung Memorial Hospital-Keelung, Keelung, Taiwan; ^3^Faculty of Biological Sciences, School of Molecular and Cellular Biology, University of Leeds, Leeds, UK; ^4^Sir William Dunn School of Pathology, University of Oxford, Oxford, UK; ^5^Department of Anatomic Pathology, Chang Gung Memorial Hospital-Linkou, Taoyuan, Taiwan; ^6^Chang Gung Immunology Consortium, Chang Gung Memorial Hospital, Chang Gung University, Taoyuan, Taiwan

**Keywords:** cytokine, EMR2, GPCR, macrophage, inflammation, signaling

## Abstract

EMR2/ADGRE2 is a human myeloid-restricted adhesion G protein-coupled receptor critically implicated in vibratory urticaria, a rare type of allergy caused by vibration-induced mast cell activation. In addition, EMR2 is also highly expressed by monocyte/macrophages and has been linked to neutrophil migration and activation. Despite these findings, little is known of EMR2-mediated signaling and its role in myeloid biology. In this report, we show that activation of EMR2 *via* a receptor-specific monoclonal antibody promotes the differentiation of human THP-1 monocytic cell line and induces the expression of pro-inflammatory mediators, including IL-8, TNF-α, and MMP-9. Using specific signaling inhibitors and siRNA knockdowns, biochemical and functional analyses reveal that the EMR2-mediated signaling is initiated by Gα_16_, followed by the subsequent activation of Akt, extracellular signal-regulated kinase, c-Jun N-terminal kinase, and nuclear factor kappa-light-chain-enhancer of activated B cells. Our results demonstrate a functional role for EMR2 in the differentiation and inflammatory activation of human monocytic cells and provide potential targets for myeloid cell-mediated inflammatory disorders.

## Introduction

Professional phagocytes such as macrophages (Mφ), neutrophils (Nφ), and dendritic cells (DCs) are critical for the recognition and elimination of invading pathogens (1, 2). The processes of phagocytosis as well as the subsequent microbial killing and immune activation/resolution by these innate immune effector cells are largely mediated *via* a diverse array of receptors and their signaling reactions (1, 3). In this regard, one receptor of interest is EMR2/ADGRE2, a human myeloid-restricted adhesion G protein-coupled receptor (aGPCR) highly homologous to F4/80, the widely acclaimed surface marker that defines murine tissue Mφ (4–6).

As a human ortholog of F4/80, EMR2 similarly contains multiple epidermal growth factor-like modules in its extracellular domain (ECD), which binds to its endogenous ligand dermatan sulfate (4, 7, 8). Initially identified as a myeloid-restricted transcript expressed in monocytes (Mos)/Mφ, Nφ, and myeloid DC (4), EMR2 protein expression was later shown to be upregulated during the *in vitro* differentiation of Mφ but downregulated following DC maturation (9). On the other hand, the strongest *in vivo* EMR2 protein signal was detected in CD16^+^ blood Mos and BDCA-3^+^ myeloid DC (10). Foamy Mφ in atherosclerotic vessels and splenic Gaucher cells are highly EMR2-positive, whereas multiple sclerosis brain foam cells express little if any EMR2 (11). The differential expression patterns of EMR2 in distinct myeloid populations strongly suggest a regulatory role of EMR2 in myeloid cell function (12, 13).

Indeed, binding and activation of EMR2 by a ECD-specific 2A1 monoclonal antibody (mAb) strongly enhanced the inflammatory responses of Nφ to a panel of stimuli, while 2A1 treatment alone (without inflammatory stimuli) did not seem effective (14). In addition, 2A1-induced EMR2 activation was shown to modulate the production of multiple cytokines and survival of lipopolysaccharide-stimulated Nφ (15). Hence, EMR2 activation seems to have a priming effect on Nφ activation. Furthermore, upregulated EMR2 expression was identified in Nφ of patients suffering from systemic inflammatory response syndrome (SIRS), and a significant association was noted between the percentage of EMR2-expressing Nφ and the extent of organ failure in SIRS patients. As a result, EMR2 was proposed recently as a novel Nφ biomarker for SIRS (14, 16). A more recent study demonstrated that Nφ of liver cirrhosis patients with infection have higher EMR2 expression levels, which showed strong correlation with disease severity and predicted overall mortality (17). Likewise, we previously showed that Mφ activated by 2A1-induced EMR2 ligation promoted secretion of several pro-inflammatory cytokines (18). More recently, a missense EMR2-C492Y variant was identified as the disease protein responsible for the autosomal dominant vibratory urticaria, a dermal vibration-induced hives. It was shown that the disease-associated EMR2 variant was less stable and prone to sensitize mast cells for aberrant histamine release upon vibratory stimulation in the presence of dermatan sulfate or 2A1 (19).

Adhesion G protein-coupled receptors represent a selective group of seven transmembrane (7TM) receptors with a large ECD that usually contains multiple tandem repeats of cell adhesion-like protein motifs and a GPCR autoproteolysis-inducing (GAIN) domain (20–22). During receptor biosynthesis, aGPCRs are normally bisected at a consensus GPCR proteolysis site *via* the GAIN domain-mediated autoproteolytic reaction into a N-terminal ECD-fragment (NTF) and a C-terminal 7TM-fragment (CTF), which remain conjugated as a dual-subunit receptor (13, 21). Recent advances indicate that aGPCR activation is likely mediated by ligand-induced NTF displacement, followed by the unfolding and binding of an internal agonist peptide to the 7TM core of CTF (23, 24). The mechanistic insights of the “tethered agonism” of aGPCRs are increasingly being unraveled, including the coupling of unique G proteins to distinct aGPCR members (21, 25–27). However, an orderly depiction of aGPCR-mediated signaling pathways is currently lacking. In the present report, we investigated and identified the involvement of Gα_16_/Akt/mitogen-activated protein kinase (MAPK)/nuclear factor kappa-light-chain-enhancer of activated B cells (NF-κB) in EMR2 receptor-mediated signaling. Our results indicate that EMR2 activation/signaling plays a functional role in the differentiation and inflammatory activation of human monocytic cells. The EMR2-induced signaling cascades reported here may help identify potential targets for the therapeutic management of inflammatory disorders, such as SIRS and vibratory urticaria.

## Materials and Methods

### Reagents and Antibodies

All chemicals and reagents were purchased from Sigma-Aldrich (St. Louis, MO, USA) unless otherwise specified. Anti-mAbs used for Western blotting against extracellular signal-regulated kinase (ERK)1/2, p-ERK1/2, p38, p-p38 (Thr180/Tyr182), c-Jun N-terminal kinase (JNK), p-JNK (Thr183/Tyr185), IκB-α, p-IκB-α (Ser32), p-Ikkα/β (Ser176/180), and p-Akt (Ser473) were obtained from Cell Signaling Technology (Beverly, MA, USA). Anti-Gα16 mAb was from Abcam (Cambridge, UK). Anti-F(ab′)2 fragment goat anti-mouse (GAM) IgG (H + L) was from Jackson ImmunoResearch (West Grove, PA, USA). Anti-CD11b-PE, anti-CD62L-PE, anti-CD81-PE, anti-CD9-APC, and anti-CD4-FITC for flow cytometry and anti-phosphotyrosine, anti-β-actin mAb for Western blotting were purchased from BD Biosciences. The mAbs used for cell stimulation were 2A1 (EMR2-specific mAb) (AbD Serotec) and mouse monoclonal IgG1 (Clone 11711) (R&D System) as described previously (18).

### Cell Culture

THP-1 (ATCC^®^TIB-202™), HL-60 (ATCC^®^CCL-240™), and U937 (ATCC^®^CRL-1593.2™) human monocytic cell lines were cultured in RPMI 1640 medium (Invitrogen) supplemented with 10% fetal bovine serum (Thermo HyClone), 1% l-glutamate, 1% penicillin, and 1% streptomycin. All cells were cultured at 37°C in a 5% CO_2_ incubator. For the induction of Mφ-like differentiation, THP-1 cells were treated with 10 nM PMA for up to 4 days. Peripheral blood mononuclear cells (PBMCs) were obtained from healthy donors’ blood by Ficoll-Plague PLUS gradient centrifugation (Amersham Bioscience, Ltd.) as described previously (18). All procedures were approved by the Chang Gung Memorial Hospital Ethics Committee (CGMH IRB No: 97-2288B) and performed according to the guideline set by the Committee. Mos were subsequently isolated from PBMCs by immunomagnetic separation using human CD14 MicroBeads MACS cell separation kit (Miltenyi Biotec, Inc.) and cultured in complete RPMI 1640 medium. When required, cells were serum starved for 16–20 h before experiment. When indicated, cell culture plates were pre-coated with appropriate mAbs (usually 10 µg/ml) in 1 × PBS at 4°C for 16 h.

For treatment with inhibitors, cells were pre-incubated with the indicated reagents at 37°C: PD98059 (20 μM/90 min) (Cayman chemical), U0126 (10 μM/60 min) (Promega), Wortmannin (10 μM/60 min) (Cayman chemical), SB203580 (5 μM/15 min) (Cayman chemical), SP600125 (10 μM/60 min) (Sigma-Aldrich), LY294002 (20 μM/30 min) (Cayman chemical), and BAY11-7082 (10 μM/30 min) (InvivoGen). For siRNA-mediated gene silencing, 200 nM of EMR2- and Gα_16_-specific siRNAs (Invitrogen) were delivered into THP-1 cells using DharmaFECT-2 Transfection reagent (GE Dharmacon) as suggested by the manufacturer and incubated for 24–48 h prior to the subsequent experiments. The sequence information of the siRNAs used is listed below: #1 EMR2-siRNA: 5′GCU CGA CUG GAA UCA GGC ACA GAA A 3′, #2 EMR2-siRNA: 5′ CAG UGA UCC CGA GAC AGA AGG UGC U 3′, #3 EMR2-siRNA: 5′ GAA CAC AAG GAU GCU GGC AUU UAA A 3′, #1 GNA15-siRNA: 5′GGC CAG AAG UCA GAG CGU AAG AAA U 3′, #2 GNA15-siRNA: 5′CCA AGA GGU UCA UCC UGG ACA UGU A 3′, #3 GNA15-siRNA: 5′ GGA CUA UCC UGG AAC UAC CCU GGU U3′.

### Cell Adhesion Assay

THP-1 cells were serum starved in RPMI medium for 16 h, harvested, and re-suspended in complete RPMI medium at 2 × 10^5^ cells/100 μl/well in a 96-well plate pre-coated with mAbs. After 1 h at 37°C, cells were washed carefully at least six times with HBSS before being fixed with 2% glutaraldehyde for 20 min at room temperature. Cells were stained with 1% methylene blue for 30 min and lysed with 100 µl of 75% ethanol after excess dye was washed off with water. Eighty microliters of lysate samples were transferred into a new ELISA plate, and absorbance was measured at OD_595_ nm.

### Flow Cytometry Assay

Cells were harvested and fixed with fresh 2% paraformaldehyde solution at 4°C for 20 min. Cells (1 × 10^6^ cells/ml) were suspended in blocking buffer (1× PBS containing 1% BSA and 5% normal goat serum) at 4°C for 1 h. Cells were subsequently incubated with appropriate concentration of first Ab in blocking buffer at 4°C for 1 h, washed thoroughly with blocking buffer, and incubated with appropriate fluorophore-conjugated second Ab (1:200 in PBS) at 4°C for 1 h. Following extensive washes with cold PBS buffer, cells were analyzed by FACScan flow cytometer (BD Biosciences). Data were analyzed using FlowJo software (Flowjo).

### Phenotypic Analysis of Neutrophil Activation

Peripheral blood Nφ were isolated from fresh venous blood donated by healthy volunteers using the Ficoll Hypaque gradient centrifugation method as described previously (15). For the morphological analysis, Nφ (5 × 10^5^ cells/ml) were incubated at 37°C for 10 min with f-MLF (1 × 10^−7^ M) or conditioned medium (CM) of THP-1 cells under various stimulated conditions as indicated. Cell images were recorded under a light microscope at a magnification of 400×. For flow cytometry analysis of the expression of cell adhesion molecules, Nφ (2 × 10^6^ cells/ml) were incubated at 37°C with the CM of THP-1 cells in the absence or presence of f-MLF (1 × 10^−7^ M) for 15 min. Cells were fixed with 2% paraformaldehyde/PBS at 4°C for immunostaining with anti-CD11b-PE or anti-CD62L-PE as described elsewhere and analyzed using CellQuest software (BD Biosciences). For the detection of reactive oxygen species (ROS) generation, Nφ (2 × 10^6^ cells/ml) were incubated with 2 µM dihydrorhodamine-123 (DHR123; Molecular Probes) or 4 mM CM-H2DCFDA (Molecular Probes) for 25 min at room temperature. Cells were then incubated for 20–30 min with the CM of THP-1 cells stimulated as indicated. The accumulation of H_2_O_2_ was immediately analyzed by flow cytometer as described (14). For chemotactic cell migration assay, Boyden chamber-type transwells (Millipore) with a polycarbonate filter of 5.0 µm pore size were employed. Nφ (5 × 10^5^ cells/ml) were seeded into the upper chambers in a total volume of 100 µl RPMI1640 containing 0.5% BSA. Relevant CM samples (400 µl) were added to the lower chambers. When necessary, f-MLF (1 × 10^−7^ M) was added in CM and used as a positive control. The transwell chamber was incubated at 37°C for 50 min, and cells migrated to the lower chambers were harvested and analyzed.

### Gelatin Zymography

Gelatin zymography assay was performed as described previously (28). In brief, serum-starved THP-1 cells (2 × 10^6^ cells/200 μl/well) were cultured in serum-free medium in 12-well plates pre-coated with or without mAbs at 37°C for 16 h. Culture medium was then collected by centrifugation at 1,500 rpm for 5 min at 4°C. Supernatant was collected and diluted 1:1 (v/v) with 2× sample buffer, heated for 30 min at 37°C before being subjected to gel electrophoresis in 8% SDS-PAGE gels containing 1 mg/ml gelatin. Following electrophoresis, gel was washed with 2.5% Triton X-100 for 10 min at RT twice, transferred into developing buffer (50 mM Tris–HCl, pH 7.4, 0.2 M NaCl, 5 mM CaCl_2_) with constant shaking at 25 rpm for 15 min at RT. Fresh developing buffer was replenished to allow for a further 48 h incubation at 37°C. Gel was subsequently transferred into fixing buffer (5% methanol, 10% acetic acid) with constant shaking at 25 rpm for 15 min at RT. Finally, gel was stained with 0.1% Coomassie brilliant blue in fixing buffer at RT, followed by destaining with fixing buffer with constant changing of the fixing buffer every 15 min until digested bands are clear.

### Cytokine Elisa Assay

Cells were seeded at 2 × 10^6^ cells/well into 12-well plates pre-coated with or without mAbs and incubated at 37°C for 16 h. After incubation, medium was collected by centrifugation at 1,500 rpm for 5 min at 4°C. Supernatant was transferred into new 1.5-ml eppendorf tubes. The levels of human IL-8 and TNF-α were measured by DuoSet^®^ ELISA Development Systems (R&D System) according to the protocol suggested by the manufacturer.

### Western Blotting Analysis

Cell lysate proteins for Western blot analysis were collected at indicated time points. In brief, cells were harvested by centrifugation at 1,500 rpm for 5 min at 4°C, washed once with ice-cold 1× HBSS, and lysed in 100-µl ice-cold modified cell lysis buffer as described previously (29). Proteins were quantified using Bicinchoninic acid protein assay kit (PIERCE, Rockford, IL, USA) by reading absorbance at 550 nm. Protein samples were separated in SDS-polyacrylamide gels by electrophoresis and transferred to polyvinylidene fluoride (PVDF) membranes (Millipore, MA, USA). Blotted PVDF membranes were blocked for 1 h in blocking buffer (5% of BSA in washing buffer) with agitation, then incubated for 1 h with the indicated first Ab (2–4 µg/ml in blocking buffer). Following extensive washes, membranes were incubated with appropriate horseradish peroxidase (HRP)-conjugated second Ab (1:2,000–1:5,000 in blocking buffer). Finally, membranes were extensively washed before detection of bound HRP by chemilluminescence (ECL, Amersham Life Science Ltd. or SuperSignal West Pico Plus, Pierce) for 5 min.

### Statistical Analysis

Quantitative analysis was performed based on results of six independent experiments unless indicated otherwise. Differences between groups were determined by Student’s *t*-test using the Prism 5 software and shown as mean ± SD. In all cases, a probability value of *p* value < 0.05 was accepted to reject the null hypothesis. The statistical significance of *p* was set at *p < 0.05, **p < 0.01, and ***p < 0.001.

## Results

### Ligation and Activation of EMR2 Receptor, a Novel Surface Marker of Mφ Differentiation, Promotes Mφ-Like Differentiation in THP-1 Cell

Consistent with our previous findings (9), EMR2 expression was indeed persistently upregulated during the *in vitro* differentiation of PMA-treated THP-1 cells, which gradually displayed characteristic Mφ-like phenotypes such as increased cell adherence, enlarged cell size and granularity, and expressional changes of specific differentiation/maturation markers including CD4, CD9, CD11b, and CD81 (Figure S1 in Supplementary Material) (30). Notably, the upregulated EMR2 expression levels correlated very closely with those of Mφ phenotypic markers. Therefore, EMR2 might be considered a novel surface marker of human Mφ differentiation.

**Figure 1:**
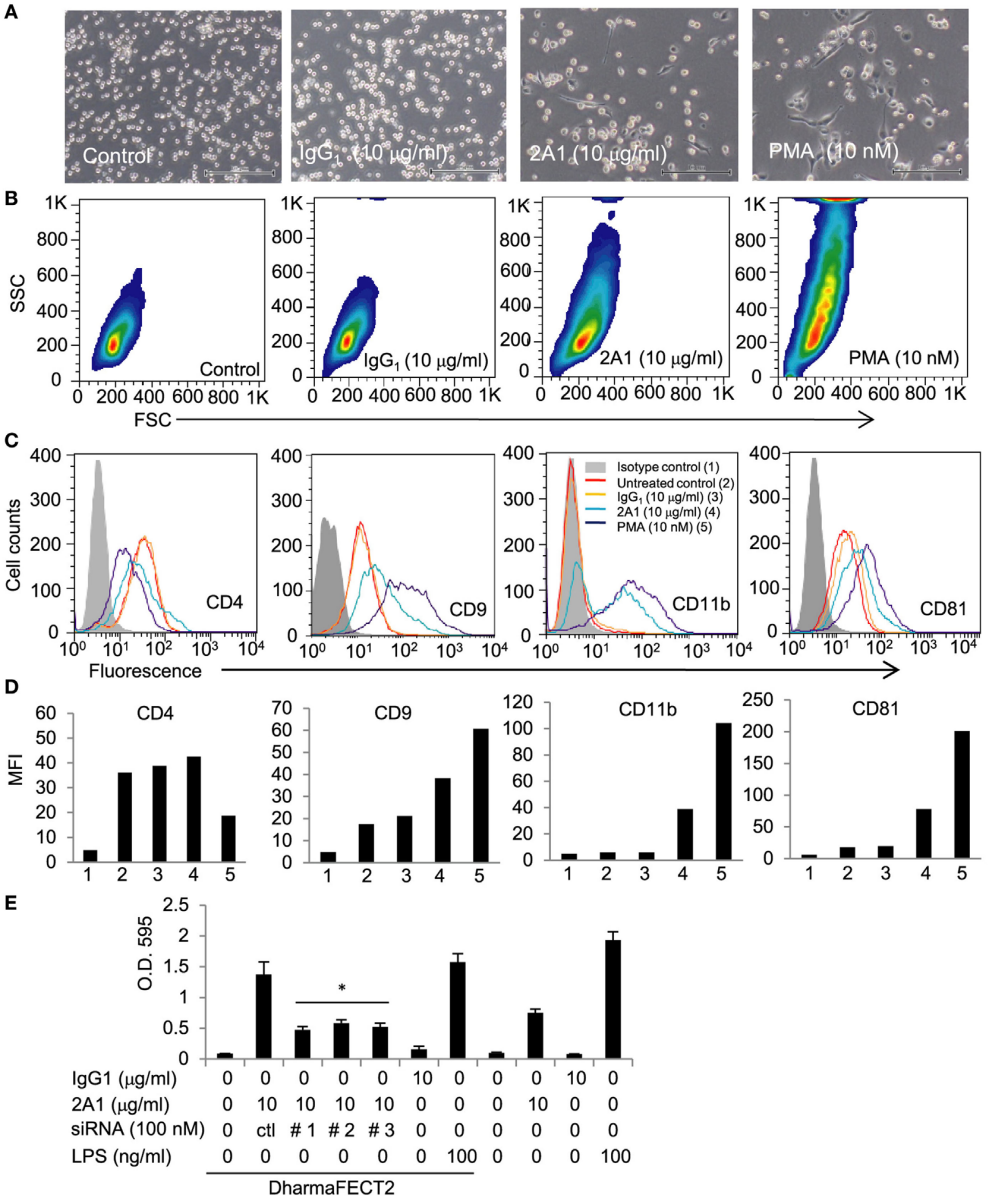
**Ligation of EMR2 receptor induces macrophage (Mφ)-like differentiation in THP-1 cell**. **(A,B)** Microscopic observation **(A)** and flow cytometry analysis **(B)** of morphological changes of THP-1 cells incubated with immobilized 2A1 monoclonal antibody (mAb) (10 µg/ml) at day 4. mIgG1 was a negative control and PMA-treated cells were a positive control. Scale bar: 10 µm. SSC: side scatter for cell granularity; FSC: forward scatter for cell size. **(C,D)** Flow cytometry analysis of specific surface marker expression of 2A1-ligated THP-1 cells shows a macrophage-like phenotype. Cells were treated with or without immobilized 2A1 mAb (10 µg/ml) for 4 days. mIgG1 was included as a negative control and PMA-treated cells were a positive control. CD4, CD9, CD11b, and CD81 were used as cell surface markers of Mφ-like differentiation of THP-1 cells. Data are one representative of three independent experiments with similar results. **(E)** The effect of EMR2 ligation and activation on THP-1 cell adhesion was evaluated by cell adhesion assay in cells transfected without or with EMR2-specific siRNAs and incubated with immobilized 2A1 mAb (*n* = 6, mean ± SD; *p < 0.05).

Interestingly, a similar Mφ-like phenotype akin to that of PMA-treated cells was detected when THP-1 cells were cultured on plates pre-coated with the EMR2-specific 2A1 mAb, which bound and ligated surface EMR2 receptor (Figure 1; Figure S2 in Supplementary Material). Importantly, no such phenotypic changes were noted in cells cultured on plates coated with control mIgG1 or in the presence of soluble 2A1 mAb (Figure 1; Figure S2 in Supplementary Material and data not shown). Finally, enhanced cell adherence induced by the immobilized 2A1 was greatly diminished in cells transfected with siRNAs that silenced EMR2 expression, confirming the specific effect of EMR2 ligation on inducing THP-1 differentiation (Figure 1E). These results indicate clearly a functional role of EMR2 activation and signaling in promoting the Mφ-like differentiation of THP-1 cells.

### EMR2 Receptor Activation in Mos Induces Inflammatory Responses

**Figure 2:**
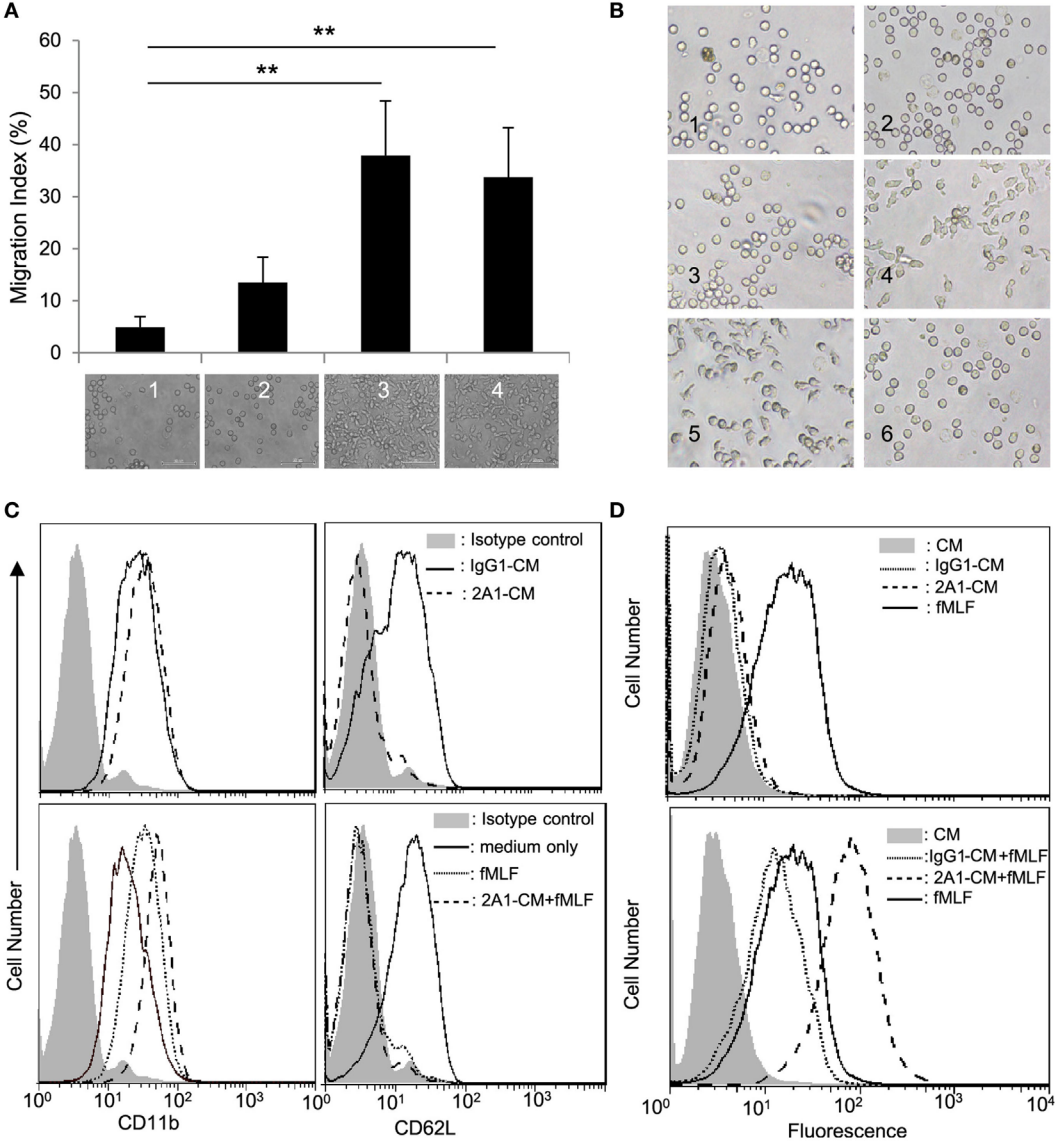
**EMR2 ligation and activation in THP-1 cells induces inflammatory responses**. **(A)** Chemotactic cell migration analysis of neutrophils (Nφ) was performed using Boyden chamber-based transwells in which the bottom wells were loaded with conditioned medium (CM) of THP-1 cells stimulated with none (THP-1 CM) (lane 1), with immobilized mIgG1 control (10 µg/ml) (IgG1-CM) (lane 2), with immobilized 2A1 (10 µg/ml) (2A1-CM) (lane 3), or THP-1 CM plus f-MLF (100 nM) (lane 4) as chemoattractants. The bottom pictures showed Nφ that have migrated across the membrane (*n* = 3, mean ± SD; **p < 0.01). **(B)** CM of 2A1-stimulated THP-1 cells induces morphological changes of Nφ. Nφ were incubated with 10 µg/ml soluble 2A1 (panel 2), 100 nM f-MLF (panel 4), or THP-1 CM (panel 1), IgG1-CM (panel 3), 2A1-CM (panel 5), or soluble 2A1 (10 µg/ml) (panel 6). Microscopic images are of 400× magnification. **(C,D)** Flow cytometry analysis of Nφ activation by detecting the expression levels of CD11b and CD62L **(C)** and ROS generation **(D)**. Nφ were treated without (top panels) or with (bottom panels) 100 nM f-MLF in the absence or presence of IgG1-CM or 2A1-CM.

Monocyte/macrophage activation usually results in dynamic changes in the surrounding milieu, further recruiting and modulating the activities of other immune cells. To explore the functional significance of EMR2 activation, CM of 2A1-ligated THP-1 cells (2A1-CM) was collected and tested for its ability to activate peripheral blood Nφ, one of the first-line innate immune effector cells. Interestingly, chemotactic cell migration assay showed that 2A1-CM was able to induce Nφ chemotaxis as well as f-MLF (Figure 2A). Cell adhesion and spreading was also observed in Nφ cultured in the presence of 2A1-CM, similar to cells treated with f-MLF (Figure 2B). By contrast, CM of THP-1 cells cultured alone or with the control mIgG1 did not induce such morphological changes in Nφ. These results strongly suggested that 2A1-CM specifically activated Nφ, which was subsequently ascertained by the identification of phenotypic changes including the upregulation of CD11b and shedding of CD62L on the cell surface (Figure 2C). Intriguingly, these phenotypic changes were more profound in Nφ under combined treatment of 2A1-CM and f-MLF than those treated singly with 2A1-CM or f-MLF, suggesting a possible synergetic effect. Moreover, it was found that while 2A1-CM treatment by itself did not activate ROS production in Nφ, combined treatment of 2A1-CM and f-MLF generated much more ROS than did cells treated with f-MLF alone (Figure 2D). Taken together, we conclude that 2A1-mediated EMR2 ligation activates THP-1 cells, which in turn induces inflammatory responses on Nφ, most likely due to the production of pro-inflammatory mediators.

### EMR2 Receptor Activation in Mos Promotes IL-8, TNF-α, and MMP-9 Production

**Figure 3:**
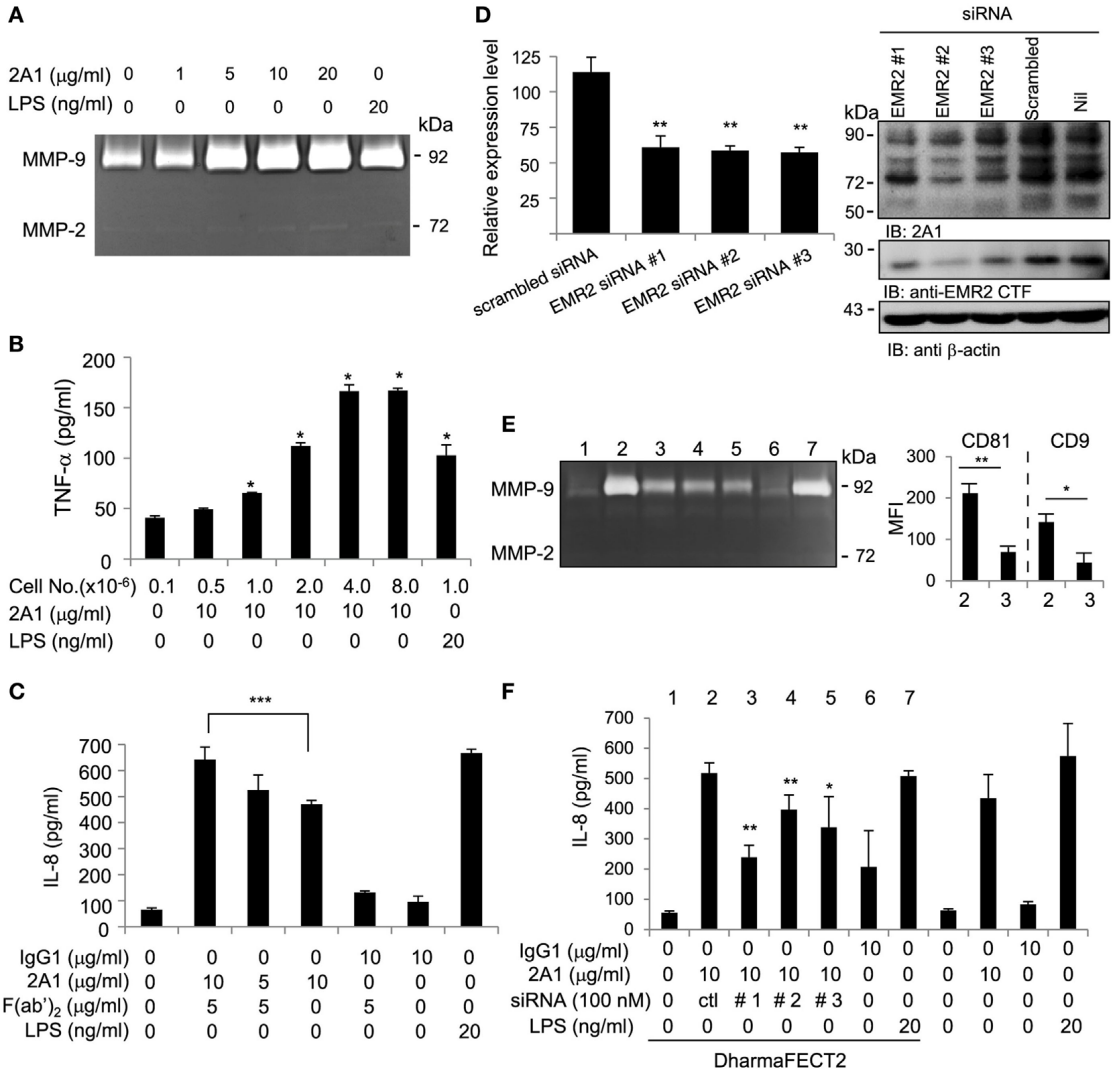
**EMR2 ligation and activation in THP-1 cells promotes IL-8, TNF-α, and MMP-9 secretion**. **(A,B)** Ligation of EMR2 on THP-1 by immobilized 2A1 mAb promoted MMP-9 and TNF-α production in a dose-dependent and cell number-dependent manner (*n* = 3, mean ± SD; *p < 0.05, **p < 0.01). **(C–F)** The specific effect of 2A1-activated EMR2 on THP-1 cells was confirmed alternatively by incubating cells at 4°C with 2A1 for 30 min followed by cross-linking with or without the F(ab′)_2_ fragment of a Goat anti-mouse Ab (5 µg/ml) as indicated. **(C)**, or by EMR2-knock down in THP-1 cells that were transfected with EMR2-specific siRNAs (EMR2-siRNA#1, #2, or #3) **(D)**. Reduced EMR2 expression was demonstrated by flow cytometry analysis of surface EMR2 levels (left panel) and Western blot analysis of lysates (right panel) of transfectant cells. The specific effect of EMR2 knockdown on THP-1 cell phenotype was shown by reduced MMP-9 production [**(E)** left panel], decreased expression of macrophage-like differentiation markers (CD9 and CD81) [**(E)** right panel], and reduced IL-8 secretion **(F)** in cells cultured on immobilized 2A1 for 4 days. Nos. 1–7 in panel **(E)** represented cell treatment conditions as listed in panel **(F)**. In all experiments, mIgG1 and lipopolysaccharide (LPS) were used as a negative and a positive control, respectively (*n* = 6, mean ± SD; *p < 0.05, **p < 0.01, ***p < 0.001).

In good agreement with our previous results (18), enhanced levels of TNF-α, IL-8, and MMP-9 were detected in 2A1-CM from THP-1 cells, as well as that of two other EMR2-expressing HL-60 and U937 cell lines (Figure 3; Figure S3 in Supplementary Material). More importantly, EMR2 ligation-induced pro-inflammatory mediator production was shown to be 2A1 dose-dependent, cell number-dependent, and time-dependent (Figures 3A,B; Figure S3B in Supplementary Material). Finally, the specificity of 2A1-induced functional effect was confirmed by two independent experiments. The first is to incubate cells first with soluble 2A1, followed by the addition of the F(ab′)_2_ fragment of a GAM Ab to cross-link receptor-bound 2A1. Interestingly, cells under this Ab-cross-linking condition also displayed similar inflammatory phenotypes as observed in those cultured with plate-bound immobilized 2A1, while cells treated with soluble 2A1 alone or GAM F(ab′)_2_ only did not (Figure 3C; Figure S3C in Supplementary Material and data not shown). The second strategy is to use THP-1 cells whose EMR2 expression was knocked down (KD) by siRNA-mediated gene silencing (Figure 3D). As shown in Figures 3E,F, EMR2-KD THP-1 cells displayed reduced MMP-9 and IL-8 production, as well as lower CD9 and CD81 levels in comparison to those transfected with a scrambled control siRNA. These results indicate that EMR2 activation cannot be achieved simply by the binding of 2A1, but requires receptor ligation (cross-linking) brought about by immobilized 2A1, which eventually leads to THP-1 cell differentiation and production of pro-inflammatory mediators.

### EMR2 Activation in Mos Induced Specific MAPK Phosphorylation

**Figure 4:**
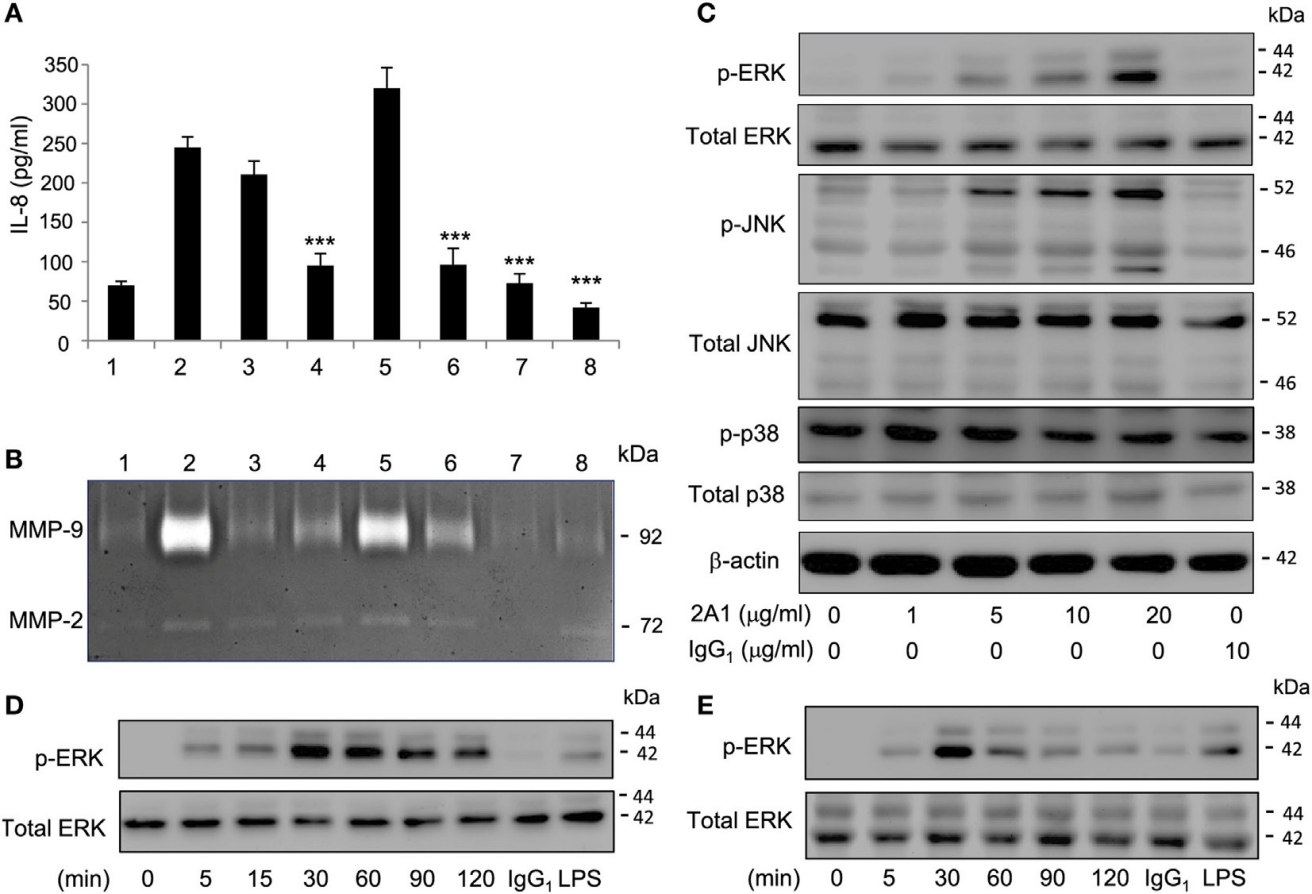
**EMR2 activation in THP-1 and primary monocytes (Mos) induced specific phosphorylation of Mitogen-activated protein kinases molecules**. **(A,B)** THP-1 cells were pretreated with different signaling inhibitors as indicated, followed by incubation with immobilized 2A1 (10 µg/ml) for 16 h. Culture supernatant was collected for the detection of IL-8 **(A)** and MMP-9 **(B)**. Lane #1, control with no 2A1 coating; #2, DMSO; #3, Wortmannin (40 µM); #4, LY294002 (50 µM); #5, SB203580 (20 µM); #6, PD98059 (20 µM); #7, SP600125 (40 µM); and #8, U0126 (10 µM) (*n* = 6, mean ± SD; ***p < 0.001). **(C)** Western blot analysis of indicated signaling molecules in THP-1 cells incubated with or without 2A1 as indicated. β-actin is a loading control for the immunoblotting. **(D,E)** Western blot analysis of EMR2 activation-induced extracellular signal-regulated kinase (ERK) phosphorylation in THP-1 cell **(D)** and primary Mos **(E)** incubated with immobilized 2A1 at the indicated time points. In all experiments, mIgG1 and lipopolysaccharide (LPS) treatment was a negative and positive control, respectively.

As an aGPCR, EMR2 activation is expected to turn on specific signaling pathways, including in principle G protein(s). To explore the EMR2-induced signaling cascades, we first examined the status of global tyrosine phosphorylation of THP-1 cell lysate following EMR2 engagement (Figure S4 in Supplementary Material). Indeed, EMR2 ligation induced fast and transient tyrosine phosphorylation of cell lysate proteins, reaching the peak at ~5 min and returned to the basal levels after ~30 min. This result clearly suggests the activation of unique signaling molecules and prompts us to survey the possible candidates using a wide range of selective signaling inhibitors. As shown in Figures 4A,B, EMR2 activation-induced IL-8 and MMP-9 production was mitigated agreeably by many inhibitors tested, including PI3K inhibitors (Wortmannin and LY294002), MAPK/ERK kinase inhibitors (PD98059, U0126), and JNK inhibitor (SP600125). By contrast, the p38 inhibitor (SB203580) in fact further enhanced IL-8 production induced by EMR2 activation. These results strongly suggest that 2A1-mediated EMR2 ligation activates multiple signaling pathways, some of which might be involved in a negative feedback loop.

As the MAPK signaling cascade is a well-known inflammation-associated signaling pathway and down stream targets of many GPCRs (31–34), we next confirmed EMR2-induced activation of specific MAPKs by Western blotting. Consistent with earlier results with the use of selective inhibitors, increased phosphorylation of ERK and JNK, but not p38, was detected following 2A1-mediated EMR2 ligation in a Ab dose-dependent and time-dependent fashion (Figures 4C–E). Importantly, EMR2 activation-induced phosphorylation of ERK and JNK was comparably detected in THP-1 cells as well as in primary blood Mos. As expected, phosphorylation of the two MAPK molecules was efficiently inhibited by specific inhibitors in a dose-dependent manner, again both in THP-1 cell and primary Mos (Figures 4D,E; Figures S5 and S6 in Supplementary Material). Simultaneously, EMR2 activation-induced pro-inflammatory mediator production was inhibited by these inhibitors (Figures S5 and S6 in Supplementary Material). We hence conclude that 2A1-mediated EMR2 ligation in Mos induces phosphorylation and activation of specific MAPK molecules, including ERK and JNK.

### EMR2-Induced Signaling Is Mediated in Part *via* NF-κB Activation

**Figure 5:**
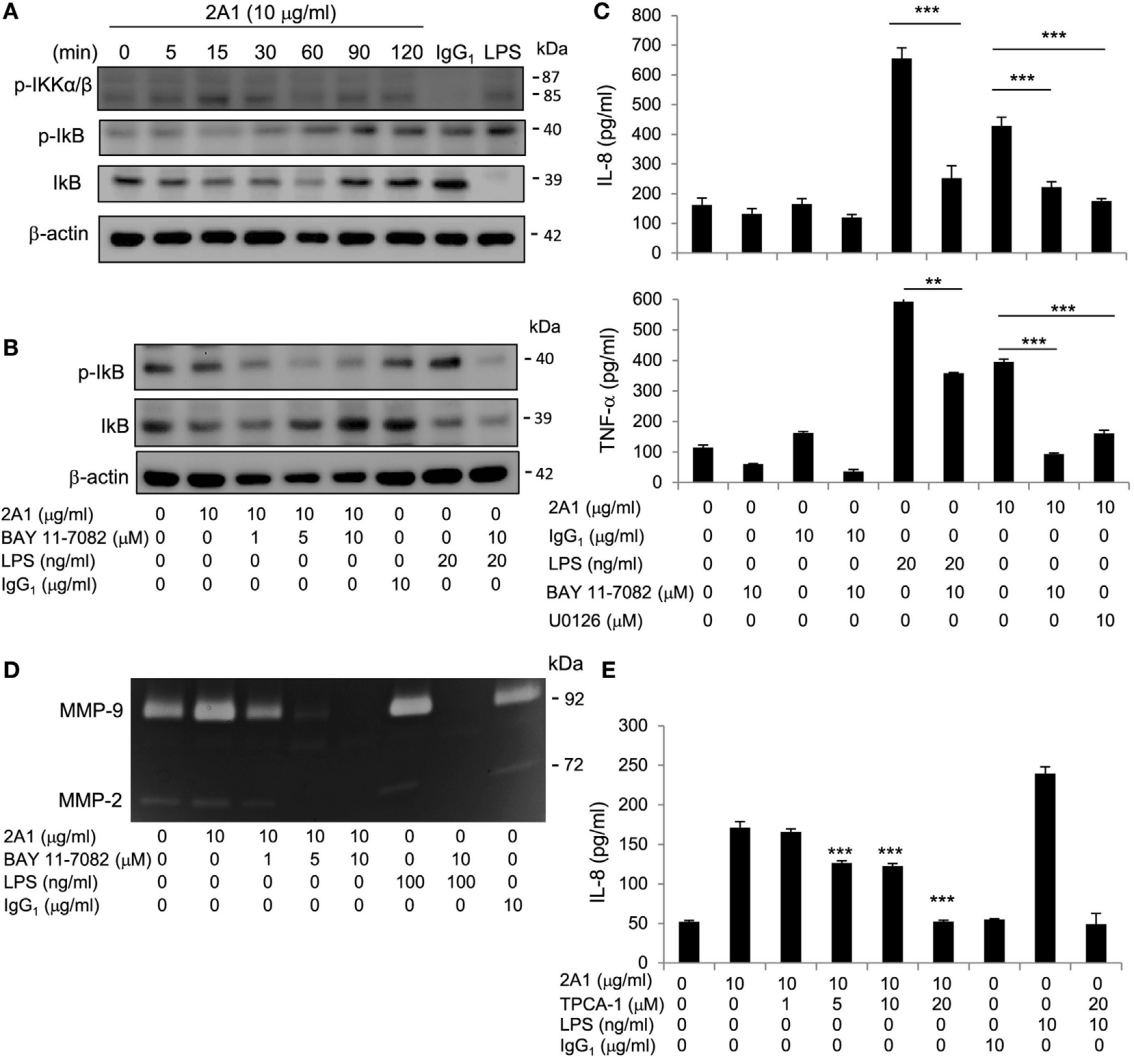
**EMR2-induced signaling in THP-1 cells is mediated in part *via* nuclear factor kappa-light-chain-enhancer of activated B cells (NF-κB) activation**. **(A)** Western blot analysis of NF-κB activation of THP-1 cells incubated with or without 2A1 (10 µg/ml) at different time points as indicated. Blots were probed to detect phospho-IKKα/β, phospho-IκB, IκB, and β-actin level. **(B)** Western blot analysis of NF-κB activation of THP-1 cells pretreated with different concentrations of Bay11-7082 for 1 h, followed by incubation with 2A1 (10 µg/ml) for 1 h. Blots were probed to detect phospho-IκB, IκB, and β-actin level. **(C,D)** Culture supernatants of THP-1 cells treated with indicated conditions for 16 h were collected for the detection of TNF-α and IL-8 by ELISA **(C)** and MMP-9 activity by gelatin zymography **(D)**. **(E)** THP-1 cells were pretreated with different concentrations of TPCA-1 for 1h, followed by incubation with 2A1 (10 µg/ml) for 16 h. Culture supernatants were collected for the detection of IL-8 by ELISA. In all experiments, mIgG1 and lipopolysaccharide (LPS) treatment was a negative and positive control, respectively (*n* = 6, mean ± SD; **p < 0.01, ***p < 0.001).

In addition to the MAPK signaling pathway, NF-κB is another major signaling molecule critical for the induction of inflammatory reactions (35, 36). In essence, NF-κB activation involves the phosphorylation and subsequent degradation of IκB, which is initiated in turn through phosphorylation of the IκB kinases (IKK) α/β (37). As shown, increased phosphorylation of IKK α/β and IκB proteins and the concurrent loss of IκB were readily detected following 2A1-mediated EMR2 ligation in THP-1 cells, again in a Ab dose-dependent and time-dependent fashion (Figure 5; Figure S7 in Supplementary Material). Furthermore, EMR2 ligation-induced NF-κB activation was specifically mitigated in THP-1 cells pretreated with two independent IKK inhibitors, namely BAY 11-7082 and TPCA-1. Coincidentally, production of IL-8, TNF-α, and MMP-9 in 2A1-stimulated THP-1 cells was dose-dependently inhibited by the treatment of BAY 11-7082 and TPCA-1 (Figure 5; Figure S7 in Supplementary Material). These data reveal clearly the induction of NF-κB signaling pathway by EMR2 ligation and activation in THP-1 cells.

### EMR2 Activation in Monocytic Cells Signals *via* the Gα_16_/PLC/Akt Pathways

Several aGPCRs have recently been shown to signal through specific G proteins (21, 38); however, very little is known for EMR2. CD97/ADGRE5, a close homolog of EMR2, was reported to heterodimerize with the lysophosphatidic acid (LPA) receptor 1 and signal *via* Gα_12/13_ to induce LPA-dependent Rho and ERK activation in prostate cancer cells (39). On the other hand, the CD97-LPAR1 heterodimer was shown to mediate the lysophosphatidylethanolamine-induced intracellular Ca^2+^ increase in MDA-MB-231 breast cancer cells by the pertussis toxin (PTX)-sensitive Gα_i/o_ protein and phospholipase C (PLC) (40). In a transient over-expression system in heterologous HEK293 cells, Gupte et al. have shown that co-transfection of EMR2 and Gα_15_ resulted in constitutive signaling (41).

**Figure 6:**
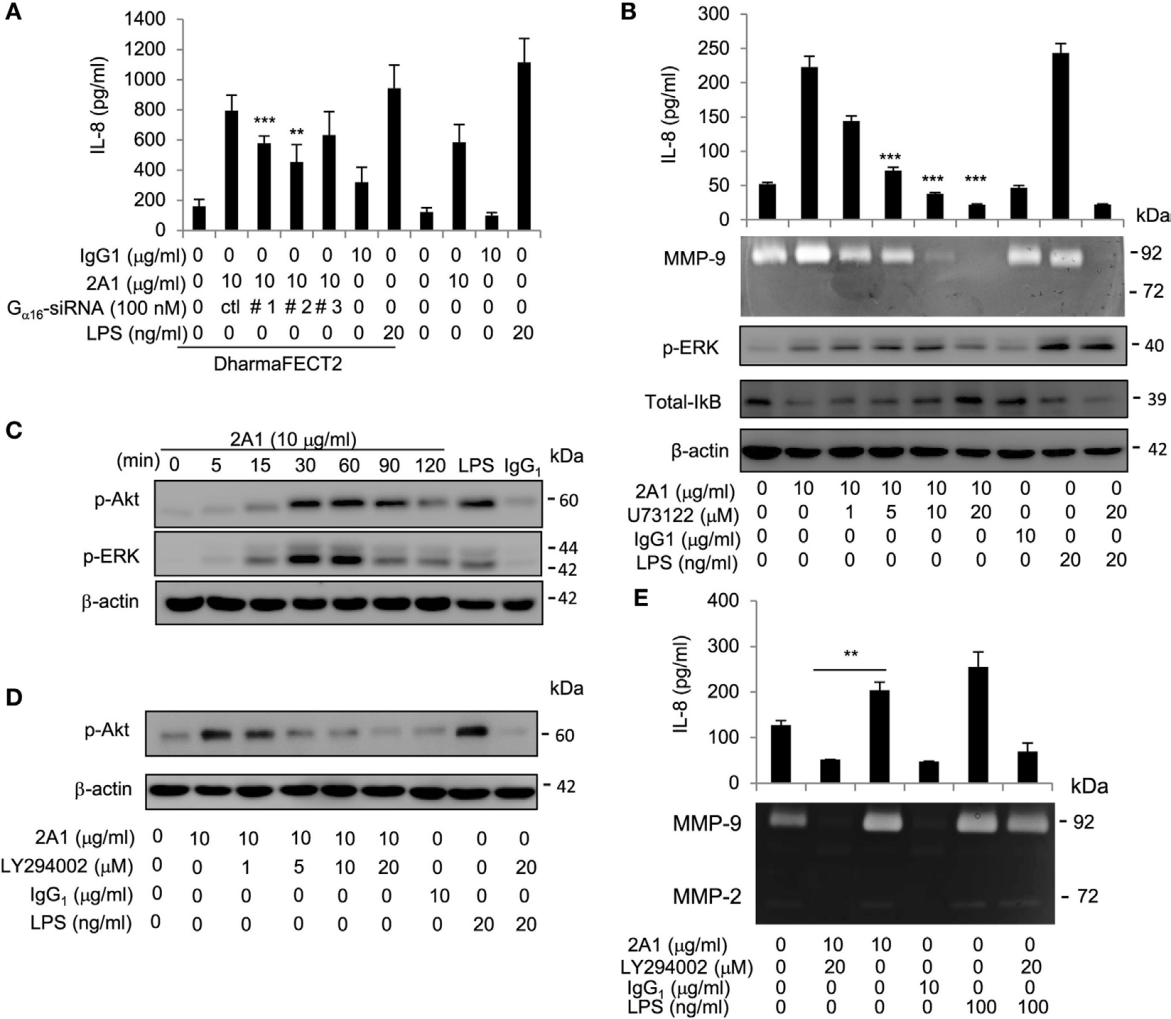
**EMR2 activation in monocytic cells signals *via* the Gα_16_/PLC/Akt pathways**. **(A)** The role of Gα_16_ in EMR2-mediated signaling was evaluated in THP-1 cells that were transfected with Gα_16_-specific siRNAs (Gα_16_-siRNA#1, #2, or #3). IL-8 levels resulted from EMR2 ligation were compared as described. **(B)** The involvement of PLC-β in EMR2-mediated signaling was evaluated in THP-1 cells that were pretreated with different concentrations of U73122 for 1 h, followed by incubation with 2A1 (10 µg/ml). Conditioned medium (CM) was analyzed for IL-8 by ELISA and MMP-9 activity by gelatin zymography, respectively (top panels). Western blot analyses were performed to detect phospho-extracellular signal-regulated kinase (ERK), IκB, and β-actin levels (lower panels). **(C,D)** Western blot analysis of EMR2 activation-induced Akt phosphorylation in THP-1 cells. Cells were pretreated without **(C)** or with different concentrations of LY294002 **(D)** for 1 h, followed by incubation with 2A1 (10 µg/ml) as shown. Blots were probed to detect phospho-Akt, phospho-ERK, and β-actin levels. **(E)** The effect of the PI3K inhibitor (LY294002) on EMR2 activation in THP-1 cells was analyzed by comparing IL-8 levels and MMP-9 activities of CM from cells pretreated without or with LY294002 as described. In all experiments, mIgG1 and lipopolysaccharide (LPS) treatment was a negative and positive control, respectively (*n* = 6, mean ± SD; **p < 0.01, ***p < 0.001).

To delineate the G protein(s) involved in EMR2-mediated signaling in monocytic cells, we first showed that PTX is ineffective in inhibiting EMR2 activation, thus excluding the involvement of Gα_i/o_ proteins (Figure S8A in Supplementary Material). Next, the role of Gα_16_ (human ortholog of murine Gα_15_) was examined using Gα_16_-specific siRNAs that effectively dampened its expression (Figure S8B in Supplementary Material). Interestingly, EMR2 ligation-induced IL-8 production was diminished when Gα_16_ expression was KD (Figure 6A), suggesting that Gα_16_ is indeed involved in EMR2-mediated signaling. Gα_15/16_ proteins are known to couple GPCRs to PLC-β, which hydrolyzes phosphatidylinositol biphosphate (PIP_2_) into inositol triphosphate (IP_3_) and diacylglycerol (42, 43). We, therefore, incubated THP-1 cells in the presence of U73122, a potent inhibitor of G protein–PLC coupling and activation. As expected, production of IL-8 and MMP-9 was inhibited dose-dependently in cells treated with U73122 (Figure 6B). In addition, diminished ERK and NF-κB activation was observed in U73122-treated cells, which is consistent with the idea that PLC-β activation is upstream of MAPK and NF-κB signaling (Figure 6B). These results show for the first time the coupling of EMR2 to PLC-β activation *via* Gα_16_.

Finally, the role of PIP_2_ metabolism by PLC-β and the fact that two PI3K inhibitors (Wortmannin and LY294002) were effective in modulating EMR2 activation (Figure 4) prompted us to investigate the involvement of Akt in EMR2-mediated signaling cascade. The PI3K-Akt pathway is initiated by activated PI3K that phosphorylated PIP_2_ to phosphatidylinositol 3,4,5 trisphosphate (PIP_3_), which then recruited and activated Akt (44, 45). Importantly, PI3K can be activated by many cell surface receptors, including GPCRs, primarily *via* dissociated Gβγ subunits (46, 47). As shown in Figure 6C, activated/phosphorylated Akt was indeed identified in THP-1 cells cultured with immobilized 2A1 in a time-dependent manner very similar to ERK activation. Moreover, Akt activation and production of IL-8 and MMP-9 were dose-dependently reduced by LY294002 in 2A1-treated THP-1 cells (Figures 6D,E), suggesting that the PI3K-Akt pathway is also involved in EMR2-mediated signaling. Taken together, we conclude that the Gα_16_/PLC/Akt pathways are coupled and activated by the ligated EMR2 receptor in monocytic cells.

## Discussion

The identification of EMR2/ADGRE2 as the vibratory urticaria-inducing molecule attests to the functional importance of this myeloid-restricted aGPCR (19). While accumulating evidence is emerging for a signaling role of EMR2 in myeloid cells, the actual pathways and significance of EMR2-mediated signaling are not understood. Herein, we show that EMR2 receptor is a novel surface marker of Mφ differentiation and EMR2 activation is mainly coupled to the Gα_16_ protein, which subsequently activates the PLC and Akt/PI3K pathways, eventually leading to the MAPK and NF-κB signaling (Figures 4–6; Figures S1–S8 in Supplementary Material). EMR2 activation promotes Mφ-like differentiation of THP-1 cells and evokes inflammatory responses by stimulating the production of pro-inflammatory mediators (Figures 1–3). In summary, EMR2 is a previously uncharacterized surface protein regulator of the differentiation/maturation process and inflammatory activation of Mφ subpopulations.

The coupling of Gα_16_ protein to EMR2 is one of a limited number of specific aGPCR–G protein partnership identified to date, but it is in line with previous results. First, it was shown recently that co-transfection of EMR2 and murine Gα_15_ in heterologous cells specifically induced cellular activation (41). Second, human Gα_16_ protein (and murine Gα_15_) are known to be restricted to cells of certain hematopoietic lineages, including myeloid cells (48). Finally, Gα_16_/Gα_15_ proteins belong to the PTX-insensitive Gα_q_ subfamily and are known to couple many GPCRs to activate PLC-β (42, 43). Due to the fact that EMR2 and Gα_16_ are co-expressing in human Mos and that EMR2 activation is impaired by siRNA-induced Gα_16_ downregulation and a pharmacological inhibitor of PLC but is unresponsive to PTX, the coupling of EMR2 activation to Gα_16_ in Mos is the most likely signaling pathway (Figure 6; Figure S8 in Supplementary Material). Nevertheless, the possibility of the involvement of other G proteins in EMR2-mediated signaling is not completely excluded.

Interestingly, the homologous CD97 receptor was shown to couple separately to Gα_12/13_ or Gα_i/o_ proteins in response to different stimuli in distinct cancer cell types (39, 40). Hence, it is possible that other Gα proteins might also be involved in EMR2-mediated signaling depending on the nature of EMR2 stimulation. Likewise, it remains to be determined whether the similar Gα_16_-mediated signaling cascades is employed in EMR2 activation in different myeloid cell types such as Nφ, mast cell, and DC.

G protein-coupled receptor-induced Akt activation is known to be mediated by all four major Gα protein subfamilies *via* distinct mechanisms (47, 49). The involvement of PI3K/Akt pathway in EMR2-mediated signaling suggested the possible participation of the dissociated Gβγ subunits and/or other means such as increased intracellular Ca^2+^ brought about by Gα_16_-induced PLC-β activity (47). Regardless of the exact mechanism, the inclusion of PLC-β/Akt-signaling pathways described here clearly indicates that phosphinositide-dependent metabolism is a significant component of EMR2-induced signaling events. This idea is further ascertained by the identification of EMR2-activated MAPKs (ERK and JNK) and NF-κB, which are validated downstream targets of the PLC-β and Akt-signaling pathways and many GPCRs (50–53).

Both MAPKs and NF-κB are well-established signaling molecules involved in the induction of inflammatory responses (31, 32, 35, 54). Thus, the activation of ERK/JNK and NF-κB by the Gα_16_/PLC/Akt pathways is consistent with the role of EMR2 in promoting Mφ-like differentiation and production of pro-inflammatory mediators. Hence, it seems that EMR2-activated signaling pathways are rather similar to the ones occurred in Mφ under inflammatory conditions. However, due to the fact that multiple signaling pathways normally cross-talk and that phagocytes usually encounter and require numerous stimuli to achieve optimal cellular activation (55), in the future it will be interesting to investigate how the EMR2-mediated signaling cross-interact and contribute to the overall Mφ activity in the presence of other stimuli.

The recent revelation of the tethered agonistic activation mechanism of aGPCRs strongly hints at the notion that some aGPCRs might function as a mechanical sensor because a physical/mechanical signal is likely needed for the dissociation of aGPCR-NTF from its CTF (38, 56, 57). Indeed, this idea was substantiated by several lines of evidences recently. These include the findings of the enhanced vibration-induced NTF-CTF dissociation of the less stable EMR2-C492Y variant in vibratory urticaria patients and the shear stress-induced shedding of CD97-NTF from leukocytes that interacted with its CD55 ligand (19, 58). While we did not directly address this issue in the present study where all experiments were performed in a static condition, it is interesting to note that the 2A1-induced EMR2 activation only occurs when the mAb is immobilized, presumably to provide enough contact/interaction to cross-link the receptor. In the future studies, it will be interesting to examine whether EMR2-NTF is dissociated from cell surface under this static condition or alternatively to test whether vibration can further enhance 2A1-induced EMR2 signaling in Mos. It will be also of interest to know whether the EMR2-C492Y variant induces stronger and/or faster signaling in Mos, apart from enhancing mast cell degranulation. Taken together, our systematic analysis of EMR2-mediated signaling not only provides a direct evidence for its role in the differentiation and inflammatory reaction of Mos but also a starting point to investigate the activation mechanism and possible manipulation of aGPCR function in diseases, such as SIRS and vibratory urticaria.

## Author Contributions

K-YI, Y-SH, C-HH, W-YT, C-HC, and G-WC performed experiments and analyzed data. MS, SG, and H-HL helped in analysis and interpretation of the data. K-YI, Y-SH, and H-HL wrote the manuscript.

## Conflict of Interest Statement

The authors declare that the research was conducted in the absence of any commercial or financial relationships that could be construed as a potential conflict of interest.

## Abbreviations

aGPCR, adhesion GPCR; CM, conditioned medium; CTF, C-terminal fragment; DAG, diacylglycerol; DC, dendritic cell; ECD, extracellular domain; EMR2, EGF-like module-containing mucin-like hormone receptor-like 2; ERK, extracellular signal-regulated kinase; f-MLF, N-formyl-methionyl-leucyl-phenylalanine; GAIN, GPCR autoproteolysis-inducing; GPCR, G protein-coupled receptor; GPS, GPCR proteolysis site; IP_3_, inositol triphosphate; JNK, c-Jun N-terminal kinase; LPA, lysophosphatidic acid; LPE, lysophosphatidylethanolamine; LPS, lipopolysaccharide; Mφ, macrophage; mAb, monoclonal antibody; MAPK, Mitogen-activated protein kinases; MMP, matrix metalloproteinases; Mo, monocyte; Nφ, neutrophil; NF-κB, nuclear factor kappa-light-chain-enhancer of activated B cells; NTF, N-terminal fragment; PLC, phospholipase C; PMA, phorbol 12-myristate 13-acetate; PI3K, Phosphoinositide 3-kinase; PIP_2_, phosphatidylinositol biphosphate; PIP_3_, phosphatidylinositol 3,4,5 trisphosphate; PTX, pertussis toxin; ROS, reactive oxygen species; SIRS, systemic inflammatory response syndrome; TNF, tumor necrosis factor; 7TM, seven transmembrane.
