# Supplementary material for: Activation of Adhesion GPCR EMR2/ADGRE2 Induces Macrophage Differentiation and Inflammatory Responses via Gα16/Akt/MAPK/NF-κB Signaling Pathways
Source: Front Immunol. 2017 Apr 3;8:373. doi: 10.3389/fimmu.2017.00373 (PMC5376562; doi:10.3389/fimmu.2017.00373)
Supplement: Supplementary file 1 [file Presentation_1.pdf]

Supplementary Material

Activation of adhesion GPCR EMR2/ADGRE2 induces macrophage differentiation and inflammatory responses via $G\alpha_{16}$/Akt/MAPK/NF-κB signaling pathways

Kuan-Yu I[1], Yi-Shu Huang[1,#], Ching-Hsun Hu[1], Wen-Yi Tseng[2,#], Chia-Hsin Cheng[1], Martin Stacey[3], Siamon Gordon[1,4], Gin-Wen Chang[1], Hsi-Hsien Lin[1,5,6,*]

*** Correspondence:** Dr. Hsi-Hsien Lin, Department of Microbiology and Immunology, College of Medicine, Chang Gung University, 259 Wen-Hwa 1st Road, Kwei-San, Tao-Yuan, Taiwan. Phone:[886]-(0)3-2118800-ext3321.Fax:[886]-(0)3-2118469. Email:hhlin@mail.cgu.edu.tw

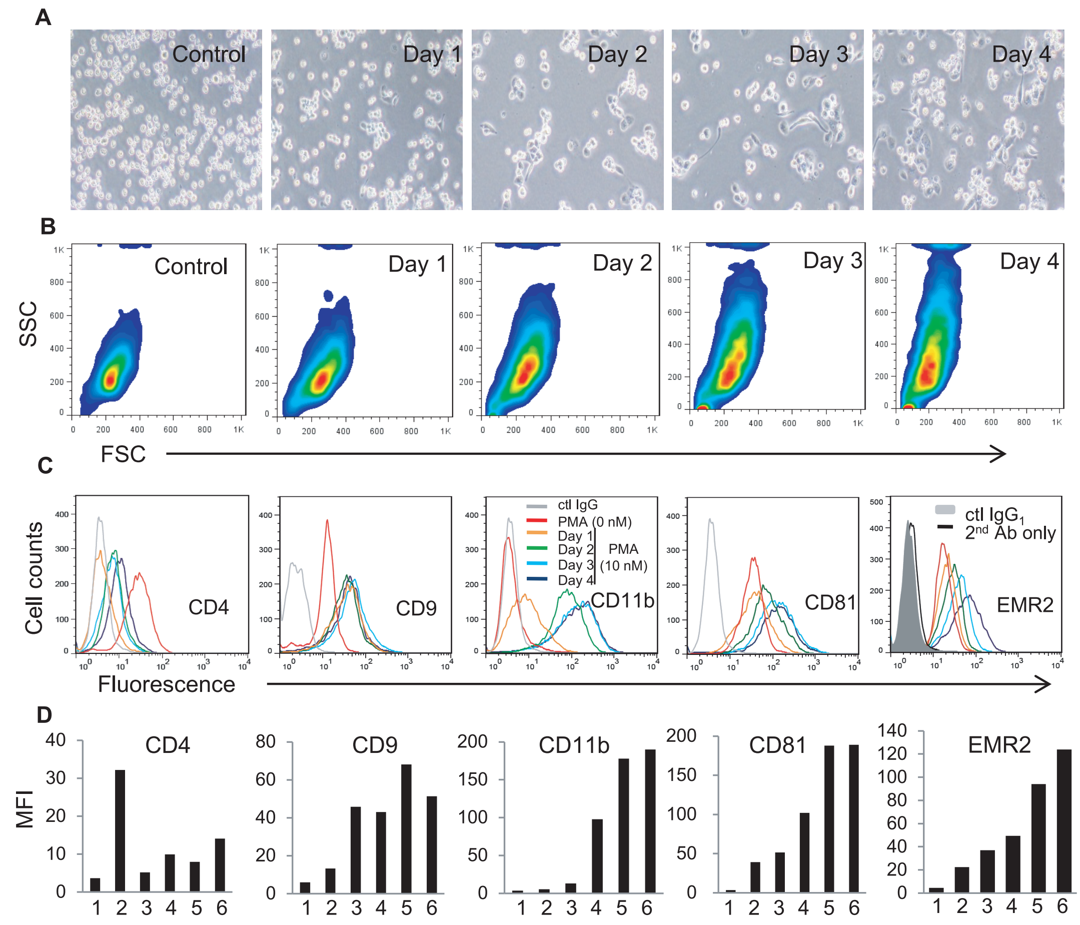

Fig. S1. EMR2 expression is upregulated during monocyte differentiation (**A**) Microscopic analysis of cell morphology and adherence of THP-1 cells treated with 10 nM PMA to promote macrophage-like differentiation at the indicated time points. Scale bar: 10 μm. (**B**) Flow cytometry analysis of morphological changes of differentiated THP-1 cells using the side scatter (SSC: for cell granularity) and forward scatter (FSC: for cell size). (**C, D**) Flow cytometry analysis of specific surface markers (CD4, CD9, CD11b, CD81 and EMR2) of PMA-induced macrophage-like THP-1 cells at indicated time points. MFI was shown for the quantitative comparison of the expression levels of indicated markers (**D**). Lane 1: IgG1 as a negative control for flow cytometry; lane 2: untreated cell control; lanes 3-6: cells treated with 10 nM PMA for 1, 2, 3 and 4 days, respectively. MFI: mean fluorescence intensity.

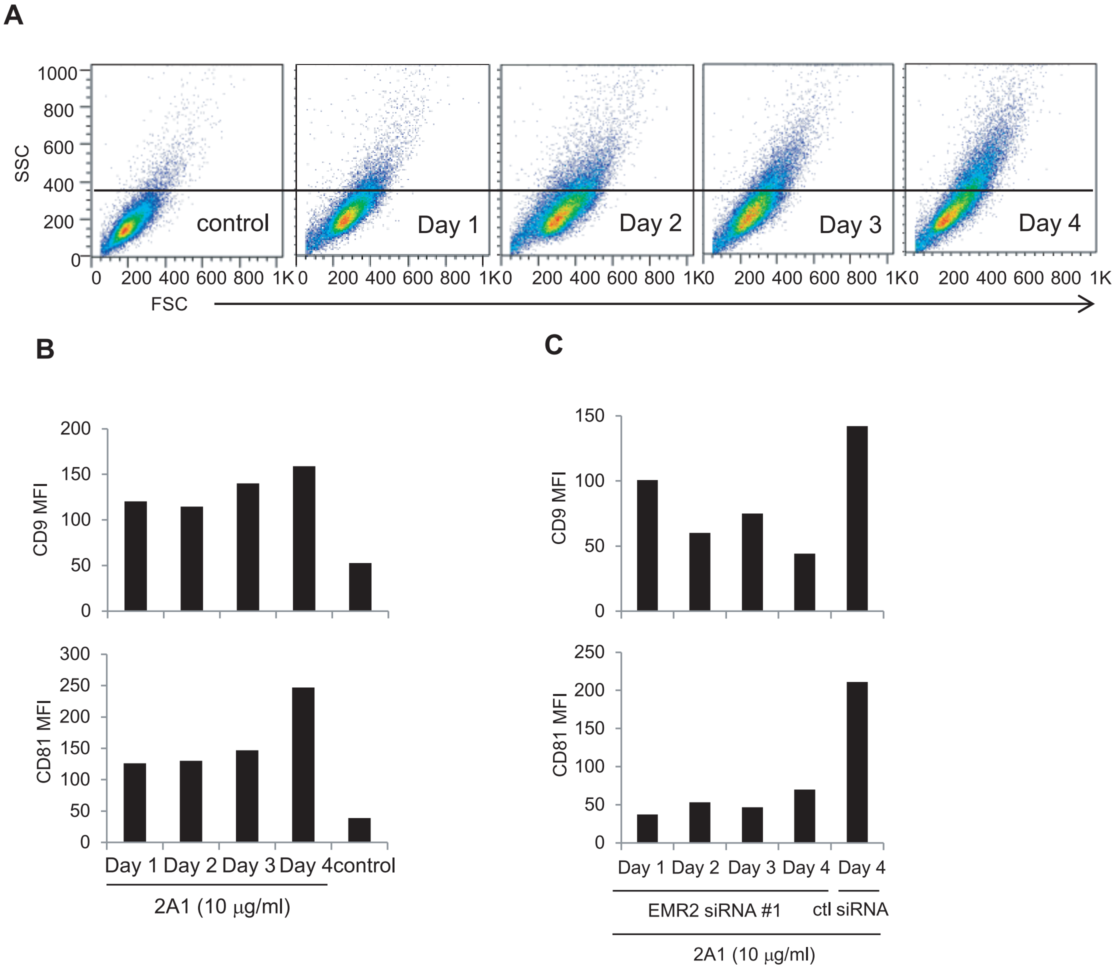

Fig. S2. Ligation and activation of EMR2 induced Mφ-like differentiation of THP-1 cells (**A**) Flow cytometry analysis of morphological changes of THP-1 cells incubated with plate-bound immobilized 2A1 mAb up to 4 days. SSC: side scatter for cell granularity; FSC: forward scatter for cell cell size). (**B, C**) Flow cytometry analysis of the expression levels (MFI) of CD9 and CD81 surface markers in THP-1 cells incubated with plate-bound immobilized 2A1 mAb up to 4 days. Cells were transfected without (**B**) or with EMR2-specific siRNAs (**C**). Data were from one representative of three independent experiments with similar results.

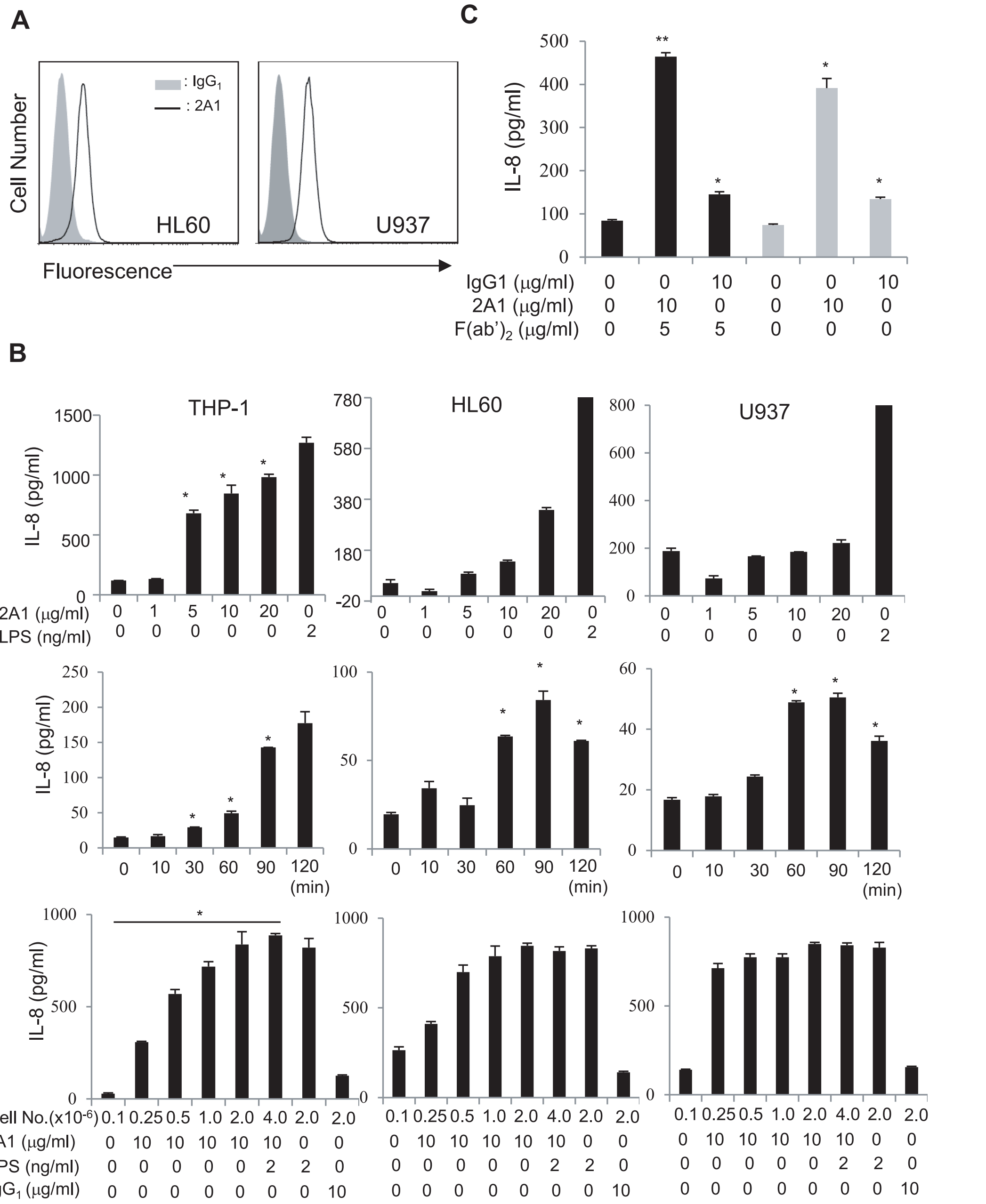

Fig. S3 EMR2 ligation and activation in human monocytic cell lines induces IL-8 secretion (**A**) Flow cytometry analysis of surface EMR2 expression in HL60 and U937 monocytic cell lines. (**B**) Ligation of EMR2 on THP-1, HL60 and U937 monocytic cell lines by immobilized 2A1 mAb promoted IL-8 production in a dose-dependent (top panel), time-dependent (middle panel) and cell number-dependent (bottom panel) manner. (**C**) EMR2 ligation and activation on THP-1 cells induced IL-8 production when cells were incubated at 4°C with 2A1 for 30 min followed by cross-linking with or without the $F(ab')_2$ fragment of a goat anti-mouse (GAM) Ab (5 μg/ml) as indicated. mIgG1 and GAM $F(ab')_2$ only were included as controls. (n=5, mean ± SD; $^{*}p<0.05$, $^{**}p<0.01$).

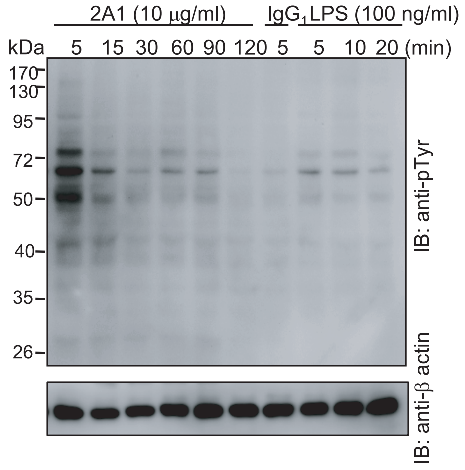

Fig. S4 Effect of EMR2 engagement on tyrosine phosphorylation in THP-1 cell
Western blot analysis of the phosphotyrosine levels on total cell lysate of THP-1 cells incubated with immobilized 2A1 (10 μg/ml) for the designated time points. mIgG1 (10 μg/ml) and LPS treatment was included as a negative and positive control, respectively

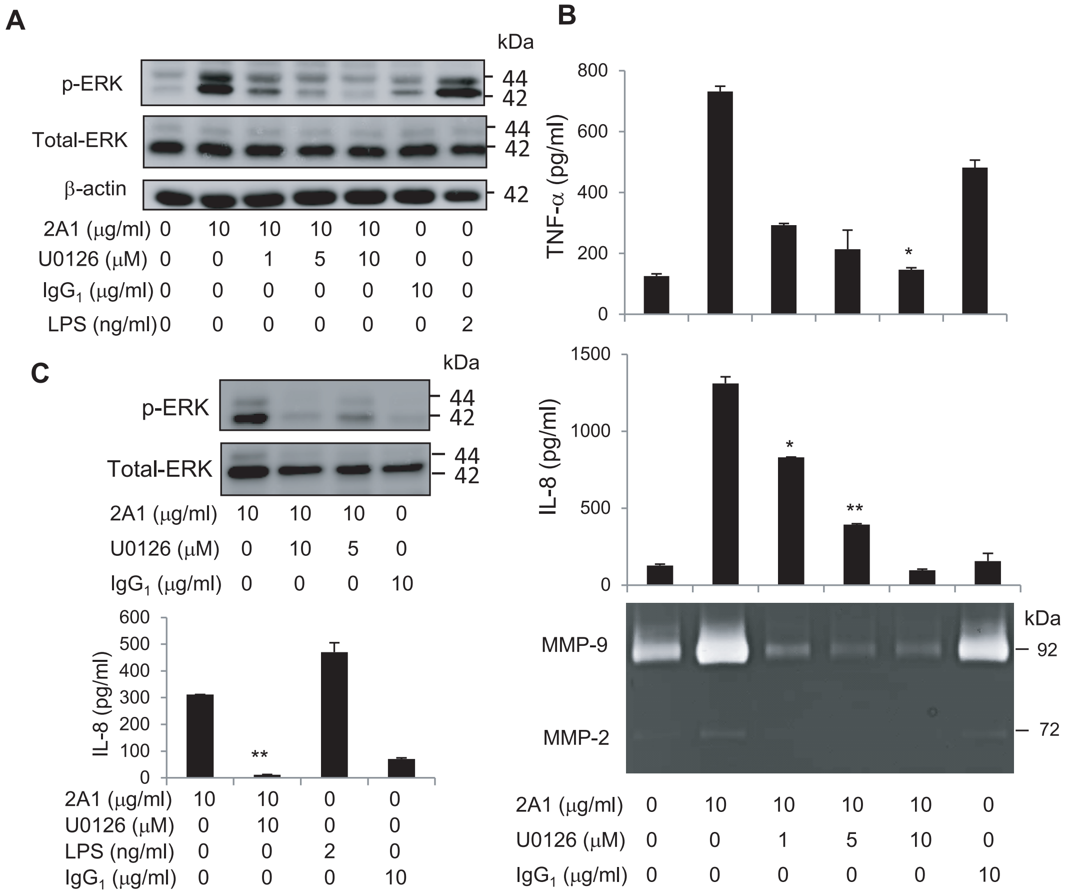

Fig. S5 EMR2-activated signaling in THP-1 cells induced specific ERK phosphorylation (**A**) Western blot analysis of ERK phosphorylation of THP-1 cells incubated with or without 2A1 for 1 hr. Cells were pretreated with or without U0126 (0, 1, 5, 10 μM). Blots were probed with primary Abs against phospho-ERK, total ERK and β-actin individually. (**B**) Culture supernatants of THP-1 cells treated with indicated conditions for 16 hr were collected for the detection of TNF-α and IL-8 by ELISA and MMP-9 activity by gelatin zymography. (**C**) Detection of ERK phosphorylation and IL-8 secretion as a result of EMR2 activation in primary monocytes incubated with 2A1. In all experiments, mIgG1 and LPS treatment was a negative and positive control, respectively. (n=6, mean ± SD; $^{*}p<0.05$, $^{**}p<0.01$).

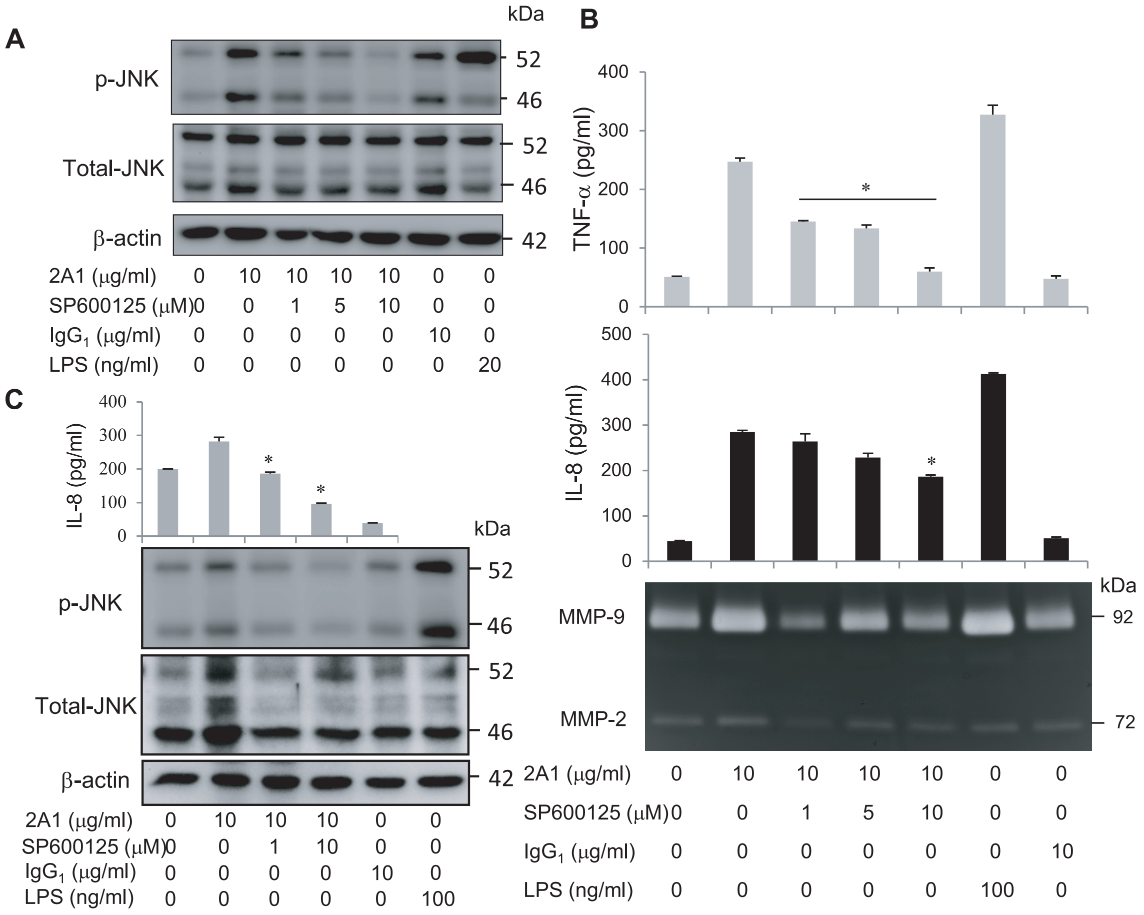

Fig. S6 EMR2-activated signaling in THP-1 cells induced specific JNK phosphorylation (**A**) Western blot analysis of JNK phosphorylation of THP-1 cells incubated with or without 2A1 for 1 hr. Cells were pretreated with or without SP600125 (0, 1, 5, 10 μM). Blots were probed with primary Abs against phospho-JNK, total JNK and β-actin individually. (**B**) Culture supernatants of THP-1 cells treated with indicated conditions for 16 hr were collected for the detection of TNF-α and IL-8 by ELISA and MMP-9 activity by gelatin zymography. (**C**) Detection of JNK phosphorylation and IL-8 secretion as a result of EMR2 activation in primary monocytes incubated with 2A1. In all experiments, mIgG1 and LPS treatment was a negative and positive control, respectively. (n=5, mean ± SD; $^{*}p<0.05$, $^{**}p<0.01$).

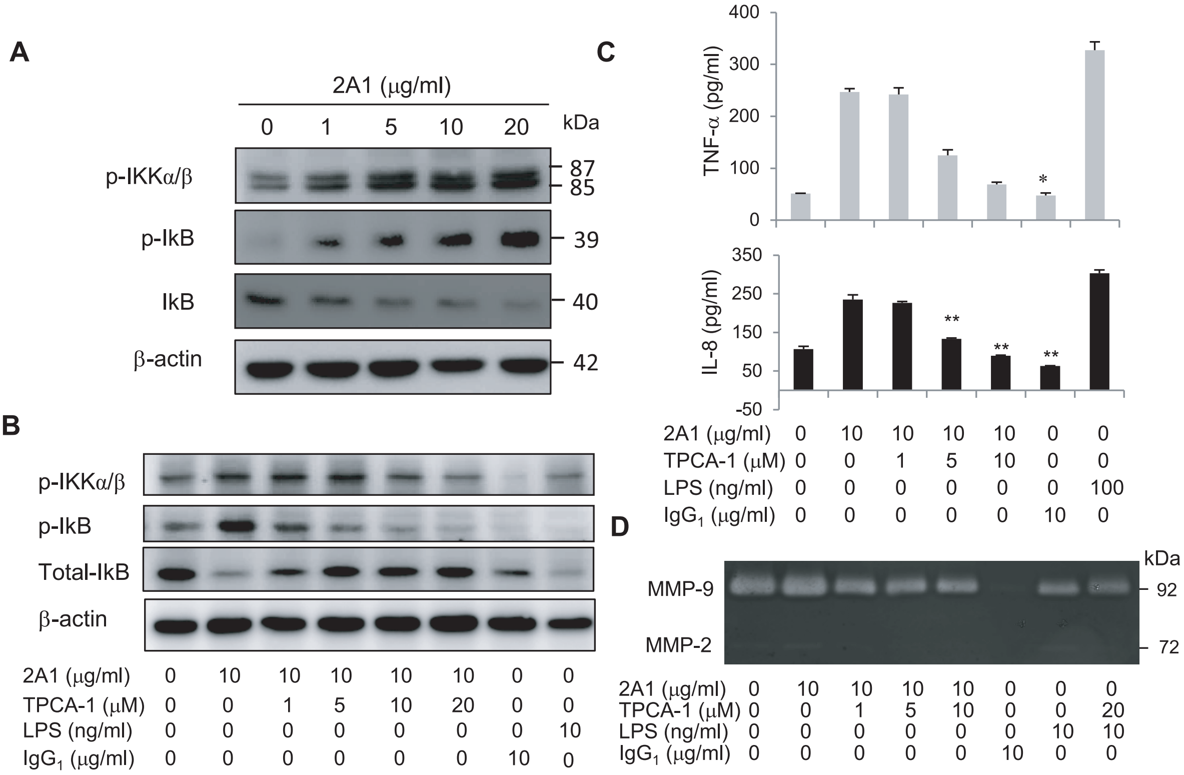

Fig. S7 NF-κB activation is involved in EMR2-induced activation in THP-1 cells (**A**) Western blot analysis of NF-κB activation of THP-1 cells incubated without or with 2A1 as indicated. Blots were probed to detect phospho-IKKα/β, phospho-IκB, total IκB and β-actin levels. (**B**) Western blot analysis of NF-κB activation of THP-1 cells pretreated with different concentrations of TPCA-1 for 1 hr, followed by incubation with 2A1 (10 μg/ml) for 1 hr. Blots were probed to detect phospho-IκB, total IκB and β-actin levels. (**C, D**) Culture supernatants of THP-1 cells treated with indicated conditions for 16 hr were collected for the detection of TNF-α and IL-8 by ELISA (**C**) and MMP-9 activity by gelatin zymography (**D**). In all experiments, mIgG1 and LPS treatment was a negative and positive control, respectively. (n=6, mean ± SD; $^{*}p<0.05$, $^{**}p<0.01$).

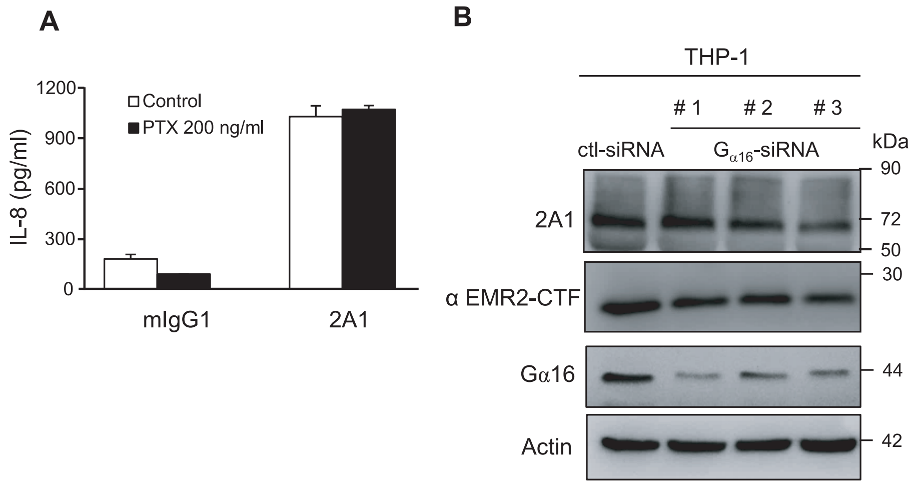

Fig. S8 Investigation of the role of G proteins in EMR2 activation in THP-1 cells (**A**) THP-1 cells were treated with or without PTX (200 ng/ml) and incubated with plate-bound mAbs as indicated for 16 hr. Culture supernatants were collected for the detection of IL-8 by ELISA. (**B**) Western blot analysis of reduced $G\alpha_{16}$ expression of THP-1 cells transfected with indicated siRNAs. Blots were probed to detect the EMR2 by 2A1 mAb and CTF-specific polyclonal Ab and $G\alpha_{16}$ by anti-$G\alpha_{16}$, respectively.
